# Correlation of Serum CCL3/MIP-1α Levels with Disease Severity in Postmenopausal Osteoporotic Females

**DOI:** 10.4274/balkanmedj.2017.1165

**Published:** 2018-07-24

**Authors:** Hui Wan, Tie-Yong Qian, Xiao-Jing Hu, Ci-You Huang, Wei-Feng Yao

**Affiliations:** 1 Department of Endocrinology, Nanjing Medical University Affiliated Wuxi Second Hospital, Wuxi, China

**Keywords:** CCL3, Chemokine, osteoporosis, post-menopausal, severity

## Abstract

**Background:**

The pro-inflammatory protein chemokine cytokine ligand 3 is well established as a vital regulator of bone resorption and osteoclast stimulation.

**Aims:**

To investigate if serum cytokine ligand 3 levels correlated with disease severity in postmenopausal osteoporotic women.

**Study Design:**

Cross-sectional study.

**Methods:**

Eighty-two postmenopausal osteoporotic women, 76 postmenopausal non-osteoporotic women, and 80 healthy women of childbearing age were recruited. The total hip, femoral neck, and L1-L4 spine bone mineral density were assessed by dual-energy X-ray absorptiometry. Serum cytokine ligand 3 concentrations were examined using a commercial enzyme-linked immunosorbent assay kit. Serum inflammatory cytokine interleukin-6, tumor necrosis factor-alpha, and the bone metabolic markers, carboxy-terminal crosslinked and tartrate-resistant acid phosphatase 5b were also examined. Scores on both the visual analogue scale and the Oswestry Disability Index were utilized to assess clinical severity.

**Results:**

Patients in the postmenopausal osteoporotic group had significantly increased serum cytokine ligand 3 levels compared with those in both the postmenopausal non-osteoporotic group (40.9±15.1 pg/mL vs 24.2±8.7 pg/mL, p<0.001) and control group (40.9±15.1 pg/mL vs 23.9±9.1 pg/mL, p<0.001). Serum cytokine ligand 3 levels negatively correlated with bone mineral density at the total hip (r=-0.345, p=0.002), femoral neck (r=-0.329, p=0.003), and L1-L4 lumbar spine (r=-0.354, p=0.001) and positively correlated with visual analogue scale scores (r=0.413, p<0.001) and the Oswestry Disability Index (r=0.360, p<0.001). Moreover, serum cytokine ligand 3 levels were correlated with increased tumor necrosis factor-alpha (r=0.305, p=0.005), interleukin-6 (r=0.288, p=0.008), terminal crosslinked and tartrate-resistant acid phosphatase 5b (r=0.371, p<0.001), and carboxy-terminal crosslinked (r=0.317, p=0.004) levels. All correlations were still significant after adjusting for both body mass index and age.

**Conclusion:**

Chemokine cytokine ligand 3 may be a useful biomarker that can be used to predict disease severity of postmenopausal osteoporosis. Therapies targeting cytokine ligand 3 and its related signaling pathways to inhibit and delay the osteoclastogenesis process deserve further investigation.

The hallmark of postmenopausal osteoporosis (PMOP) is rapidly deteriorating bone volume experienced during the postmenopausal period (1). This condition is emerging as a significant metabolic bone disease, given the global demographic shift toward an increasingly aged society. Studies estimate that nearly 200 million people around the world suffer from osteoporosis (OP), with approximately 8.9 million people experiencing osteoporotic fractures (2). Although the etiology of OP remains unclear, lack of estrogen, malnourishment, and several genetic factors have been found to contribute to the development of OP (3).

On the level of cellular pathophysiology, there is an imbalance between bone resorption and new bone formation by osteoclasts and osteoblasts, respectively, resulting in an overall reduction in bone mass. Usually, OP results from a preponderance of osteoclast activity over that of osteoblasts (4). Bone loss is much more common in trabecular bone since it undergoes more extensive bone remodeling than cortical bone does (5). Therefore, bones with a higher proportion of trabecular bone, including the vertebrae and femoral neck, are more susceptible to OP (6).

So far, OP is mainly diagnosed based on the complaints of back pain, radiographic changes of bone volume, and bone mineral density (BMD) at both the proximal femur and lumbar spine (7). However, radiographic alterations including the presence of bone loss are usually signs of medium-late stage OP (8). Recently, biochemical markers involved in increased bone turnover have been proposed as potential indicators of the degree of severity of bone resorption (9). Accumulating evidence supports markers that indicate bone turnover to be associated with OP progression. Therefore, these markers are being investigated as biomarkers for diagnosing early OP and for monitoring disease progression (10).

Chemokines are small, soluble chemo-attractive cytokines involved in regulating cell recruitment. They have been implicated in cell differentiation, apoptosis, and proliferation, as well as other physiological activities (11). Chemokine cytokine ligand 3 (CCL3), also known as macrophage inflammatory protein-1α is one of the most extensively investigated chemokines. CCL3 is a chemo-attractive cytokine chemokine that has chemotactic activity on dendritic cells, monocytes, basophils, and eosinophils. In recent years, the role of CCL3 in bone metabolism has been partially investigated. CCL3 binds to its receptor, chemokine cytokine receptor 1, to inhibit the differentiation, proliferation, and osteogenic potential of osteoblasts by impairing mineralization activation by downregulating the expression of osteocalcin (OCN), runt-related transcription factor 2 (Runx2), and osterix (Osx) (12). Moreover, administering an anti-CCL3 antibody can increase the levels of OCN, Runx2, and Osx, partially restoring the activity of osteoblasts (12).

CCL3 also functions as a chemotactic molecule for mature osteoclasts (13) and osteoclast precursor cells (14). Rat bone marrow cultures display elevated levels of osteoclasts upon exposure to CCL3 (15,16). Experiments also (17) implicated CCL3 in multiple myeloma, a condition characterized by pathological osteoclastogenesis.

Taken together, it is possible that CCL3 will play a pivotal role in OP progression. Nevertheless, the current literature does not fully explain the relationship between serum concentrations of CCL3 and OP disease severity. Hence, in this study, we sought to quantify serum CCL3 levels in patients with OP to evaluate its utility as a biomarker for predicting PMOP disease severity.

## MATERIALS AND METHODS

This study was approved by Nanjing Medical University Affiliated Wuxi Second Hospital Scientific or Ethical Committee (approval number: 2016-05). From September 2016 to June 2017, 82 postmenopausal women diagnosed with OP (PMOP) in our hospital were recruited into the current cross-sectional study. All diagnoses were based on the World Health Organization diagnostic criteria for OP (18). Exclusion criteria were as follows: the presence of other metabolic bone diseases that may cause low BMD, such as osteomalacia, hyperparathyroidism, and vitamin D deficiency; presence of significant renal, liver, or cardiovascular disease; a history of calcitonin, selective estrogen receptor modulators, or estrogen intake within the last 3 months or a history of bisphosphonates (BP) or glucocorticoids intake within the last 6 months. Seventy-six postmenopausal women with normal bone volume [postmenopausal non-osteoporotic (PMNOP)] and 80 healthy women of childbearing age (ranging from 20 years to 49 years) were recruited as controls. All participants were informed of the study process and provided consent.

### Laboratory examination

At 8:00 AM after overnight fasting, venous blood was collected from all participants in vacutainer tubes and quickly centrifuged. Samples were frozen at -80 °C before the examination. Levels of CCL3 were assessed in a blinded fashion by a quantitative sandwich enzyme-linked immunosorbent assay kit (Quantikine; R&D Systems, Minneapolis, MN). Blank controls, standards, and sample wells were set according to the manufacturer’s instructions. The sample concentrations of CCL3 were calculated from the standard curve. Serum levels of interleukin (IL)-6 (1:1000, Santa Cruz, USA), tumor necrosis factor-α (TNF-α) (1:1000, Santa Cruz, USA), tartrate-resistant acid phosphatase 5b (TRACP-5b) (1:1000, Abcam, Cambridge, UK), and cross-linked carboxy-terminal telopeptide of type 1 (CTX-1) (collagen, 1:1000, Abcam, Cambridge, UK) were also tested using the same procedure. The inter-assay for CCL3, IL-6, TNF-α, CTX-1, and TRACP-5b were shown to be 3.3%, 5.2%, 4.0%, 3.1%, and 3.9%, respectively. The intra-assay coefficients of variation CCL3, IL-6, TNF-α, CTX-1, and TRACP-5b were 4.6%, 6.6%, 6.3%, 7.5%, and 5.3%, respectively. All sample tests were repeated at least three times.

### Evaluation of clinical severity

Clinical severity was evaluated using the Oswestry Disability Index (ODI) and the visual analogue scale (VAS). Scores on the VAS were evaluated using a scale ranging from 0 (no pain present) to 100 mm (worse possible pain) (19). The ODI (20) evaluates “back-specific functions” via a self-administered questionnaire assessing 10 items, with points ranging from 0 to 5 for each item and the final score totaling between 0 to 100 points. The ten items include pain intensity, sleeping, standing, sitting, walking, lifting, personal care, travel, work, and social life. Patients with higher scores have more severe disease when compared with patients with lower scores.

### Evaluation of bone mineral density

The BMD for all subjects was assessed with the help of dual energy X-ray absorptiometry (Prodigy Advance, General Electric Company, Fairfield, CT, USA) scans at the femoral neck, total hip, and lumbar (L1-L4) spine. Bone area and bone mineral content scores were used to calculate BMD (g/cm^2^). Patient scans were performed by an independent operator who was blinded to the results of the analysis.

### Statistical analysis

All data were described as mean ± standard deviation, median (interquartile range), or frequencies. Statistical normality was assessed with the Kolmogorov-Smirnov test. Comparisons of the characteristics between PMOP women, PMNOP women, and healthy controls were performed by a one-way ANOVA or Kruskal-Wallis test, depending on the data distribution. Bartlett’s test was applied to examine the group variance homogeneity with the Tukey or Tamhane test for post-hoc analysis. Correlations of CCL3 concentrations in serum with BMD, clinical severity, and biochemical indices were calculated by both Spearman correlation and multivariate linear regression analyses. The adjusted correlation coefficients by body mass index (BMI) and age were also calculated. All analyses were performed using the Graph Pad Prism 6.0. P values less than 0.05 were considered statistically significant. The statistical power was calculated by the tool, Power Analysis and Sample Size, (PASS 2008 Statistical Software, UT, USA) based on different means of CCL3 levels, standard errors, and the number of enrolled patients in each group. Strong statistical power was determined when >0.8.

## RESULTS

### Basic data of all participants

**Figure 1:**
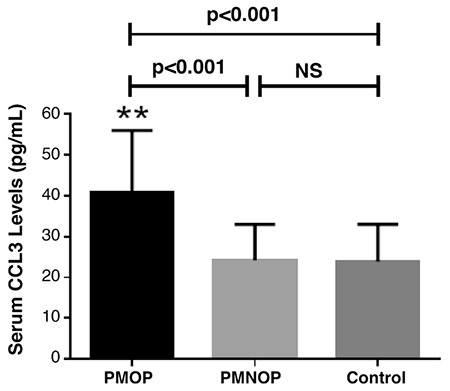
Comparison of serum chemokine cytokine ligand 3 concentrations among postmenopausal osteoporosis, postmenopausal non-osteoporotic, and control groups. **p<0.001. *CCL3: chemokine cytokine ligand 3; PMNOP: postmenopausal non-osteoporotic; PMOP: postmenopausal osteoporosis*

Table 1 depicts the demographics and clinical characteristics of all subjects. The average age was 65.8±5.1 years in the PMOP group, 64.9±5.7 years in the PMNOP group, and 44.9±5.0 in control group. No significant inter-group differences in BMI were noted in the PMOP (24.0±3.0 kg/m^2^) cases when compared with the PMNOP (23.9±3.3 kg/m^2^) and the control group (23.6±3.4 kg/m^2^). Serum CCL3 levels were significantly elevated in the PMOP group compared with those in the PMNOP group (40.9±15.1 pg/mL vs 24.2±8.7 pg/mL, p<0.001) and the control group (40.9±15.1 pg/mL vs 23.9±9.1 pg/mL, p<0.001) (Figure 1, Table 1). Calculated statistical power was determined to be 0.92.

### Correlation of serum chemokine cytokine ligand 3 levels with bone mineral density

**Figure 2:**
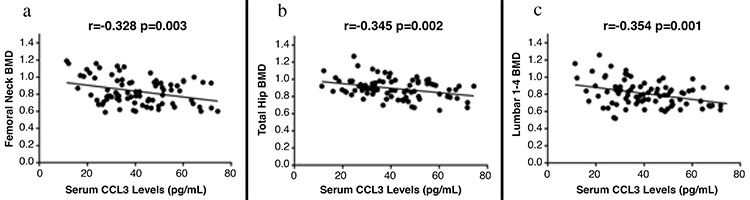
The relationship between serum chemokine cytokine ligand 3 concentrations with bone mineral density in postmenopausal osteoporosis patients. *BMD: bone mineral density; CCL3: chemokine cytokine ligand 3*

The bone mineral density at  femoral neck, L1-4 lumbar spine and total hip were significant lower in PMOP group compared with PMNOP and control group (Table 2). Serum CCL3 levels were analyzed to determine their correlation with BMD in PMOP. We found higher CCL3 levels were correlated with lower femoral neck BMD (r=-0.329, p=0.003), lower total hip BMD (r=-0.345, p=0.002) (Figure 2a) and lower lumbar 1-4 BMD (r=-0.354, p=0.001) (Figure 2c).

### Correlation of chemokine cytokine ligand 3 in serum with clinical severity and biochemical indices

**Figure 3:**
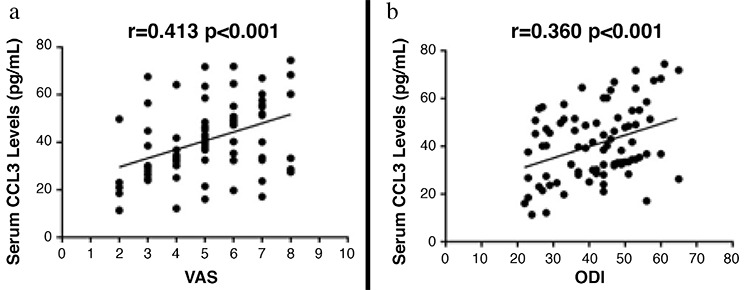
The relationship between serum chemokine cytokine ligand 3 concentrations and visual analogue scale as well as Oswestry Disability Index in postmenopausal osteoporosis patients. *CCL3: chemokine cytokine ligand 3; ODI: Oswestry Disability Index; VAS: visual analogue scale*

**Figure 4:**
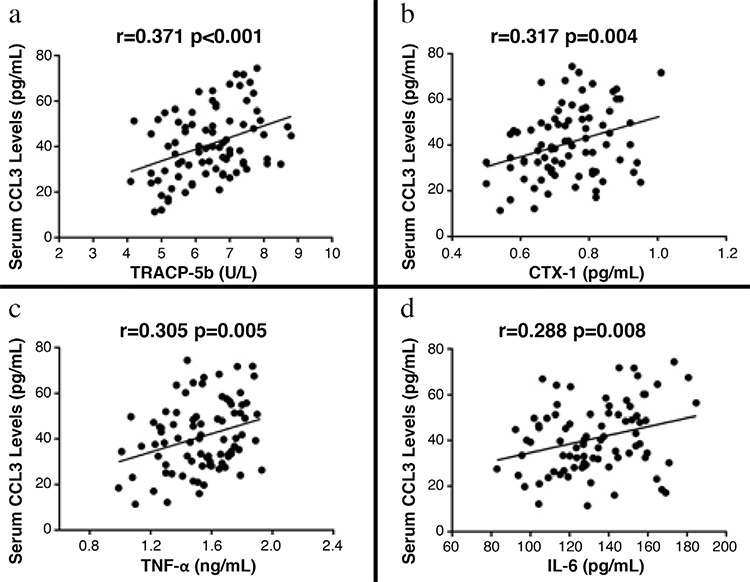
The relationship between serum chemokine cytokine ligand 3 concentrations with bone turnover markers as well as inflammatory factors in postmenopausal osteoporosis patients. *CCL3: chemokine cytokine ligand 3; CTX: carboxy-terminal telopeptide; IL: interleukin; TNF: tumor necrosis factor; TRACP: tartrate-resistant acid phosphatase*

We explored the correlation of serum CCL3 concentrations with VAS and ODI clinical severity scores to illustrate whether CCL3 is related to clinical manifestations. We also showed that CCL3 concentrations in serum were significantly related to clinical severity defined by VAS (r=0.413, p<0.001) (Figure 3a) and ODI (r=0.360, p<0.001) (Figure 3b). CCL3 was positively associated with bone resorption markers TRACP-5b (r=0.371, p<0.001) (Figure 4a),  CTX-1 (r=0.317, p=0.004) (Figure 4b) and inflammatory cytokines TNF-α (r=0.305, p=0.005) (Figure 4c) as well as IL-6 (r=0.288, p=0.008) (Figure 4d). All these correlations remain significant adjusted by age and BMI (Table 3). Multivariate linear regression analysis demonstrated that CCL3 could serve as an independent candidate marker for the assessment of both BMD and clinical severity (Table 4).

## DISCUSSION

To the best of our knowledge, our findings are the first to illustrate that women with PMOP who have decreased BMD also have increased levels of serum CCL3, demonstrating a correlation between this chemokine and OP. In addition, we found that levels of CCL3 mirror the symptomatic severity as evaluated by both VAS and the ODI. CCL3 levels were also related to the expression of biochemical indices. This data suggests that CCL3 is an important mediator of OP progression in PMOP women.

During the past few years, a number of studies have been performed to explore in detail the regulatory mechanisms for both physiological and pathological bone turnover as well as in the field of “osteoimmunology” an emerging field of research seeking to understand skeletal and immune system interactions further (21). Bone tissue and the immune system have been shown to have overlapping functions via shared receptors, soluble molecules, and signaling pathways (22). Therefore, inflammation or the immune response may play a pivotal role in the progression of OP.

Chemokines are cytokine factors that mediate both the activation and migration of leukocytes and other cells expressing G-protein coupled receptors (23). There are four major subgroups of chemokines that are based on their amino-terminal conserved cysteine residue sequences, namely CXC, CC, C, and CX3C (23). Chemokines are crucial in initiating osteoclast activation and osteoclastogenesis (24). CCL3, in particular, is induced by RANKL during osteoclast differentiation (25) and is a well-established factor that induces osteoclast formation in multiple myeloma (17). CCL3 is a chemotactic cytokine for macrophages and acts to induce osteoclasts gathering and activity further (25). Osteoblastic cells express CCL3 at bone remodeling sites (26) in association with osteoclast proximity, suggesting that CCL3 directly affects osteoclastogenesis or recruits osteoclast precursors. We demonstrated that serum CCL3 levels were associated with decreased BMD in the femoral neck, total hip, and 1^st^ to 4^th^ lumbar vertebrae, suggesting that CCL3 may both induce osteoclast activity and lead to decreased bone volume.

Chronic back pain is one of the most important complications of PMOP. Vague lower back pain is common in osteoporotic patients, irrespective of the presence of a vertebral fracture. Ohtori et al. (26) reported that up to 10.4% of PMOP patients with lower back pain had no evidence of fractures (27). In addition, decreased BMD is related to an increased risk of fracture of the vertebra, which, in turn, may also contribute to chronic pain and restricted back movement (28,29). Drugs used to treat OP including BP can alleviate pain by acting on osteoclast activity. Two clinical studies reported that OP patients without vertebral fractures treated with BP showed a significant increase in their lumbar BMD as well as relief of back pain (27,30). These findings suggest that OP, bone resorption, and pain were tightly correlated, and bone resorption may cause osteoporotic bone pain. CCL3 has been shown to modulate pain. One previous study has shown that elevated CCL3 expression in inflamed tissues is closely associated with both acute and chronic inflammatory hyperalgesia and chronic mechanical allodynia (31). In addition, levels of inducible CCL3 have been shown to be significantly increased, alongside the heightened production of inflammatory cytokines, in patients with chronic and recurrent cervical neck pain (32). In this study, we found that increased CCL3 concentrations in serum are positively related to both VAS and ODI, implying that CCL3 may participate in pain related to osteoclast activity.

Bone-resorbing osteoclasts strongly express the TRACP enzyme. Two types of TRACP exist in human blood circulation: TRACP-5a derived from dendritic cells and macrophages, and osteoclast-derived TRACP-5b (33,34). Recent studies have suggested that TRACP-5b may function as a marker of osteoclast activity, the number of osteoclasts, and bone resorption (35). CTX-1 has been shown to be a reference marker in OP for monitoring disease progression, the risk for fracture, and prognosis (36). A recent meta-analysis of six prospective studies in postmenopausal women and elderly men revealed a modest but significant correlation of serum CTX-1 and fracture risk (37). CTX-1 appears to be a sensitive, specific, and rapidly available bone resorption biomarker that can predict a PMOP patient’s response to BP therapy. Previous studies have identified that CTX-1 and TRACP-5b were significantly elevated in PMOP patients when compared with healthy controls, and were negatively correlated with BMD (38,39). In addition, estrogen deficiency during menopause disproportionally increases osteoclastic resorption activity without a corresponding increase in osteoblastic activity, resulting in a higher amount of bone resorption compared with bone deposition, and ultimately producing a net loss of bone (40). Meanwhile, estrogen levels fall and inflammatory markers such as IL-6 and TNF-α levels increase, all of which accelerate the bone remodeling cycle, with intensive activation of osteoclasts, and greater bone resorption. In this study, we discovered a positive correlation between serum CCL3 levels and elevated CTX-1 and TRACP-5b levels. We also observed a positive relationship between CCL3 and IL-6 as well as TNF-α levels, further supporting the role that CCL3 played in osteoclasts activity along with bone turnover markers.

This study has a number of limitations. First, our study is designed as a cross-sectional study that involves a relatively small sample size. Our findings would benefit from further validation by undertaking longitudinal studies in a larger cohort. Second, the concentrations of other chemokines potentially involved in OP progression were not assessed. Last, we did not explore whether drug treatment could decrease serum CCL3 levels.

In conclusion, we found that CCL3 levels were more elevated in PMOP than in healthy women. CCL3 levels were positively correlated with disease severity in PMOP. CCL3 in serum may represent a novel potential biomarker that reflects PMOP disease severity. Studies that are more extensive are warranted to evaluate the potential utility of CCL3 in monitoring both the development and progression of PMOP further.

## Figures and Tables

**Table 1:**
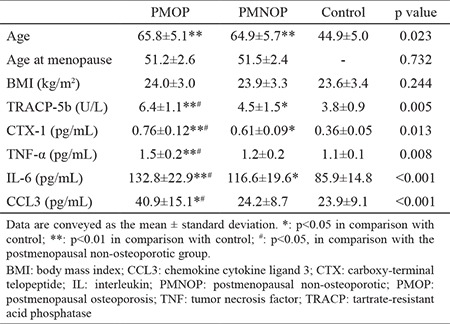
Comparison of demographic data, biochemical indices and CCL3 levels among postmenopausal non-osteoporotic, postmenopausal osteoporosis, and healthy control groups

**Table 2:**
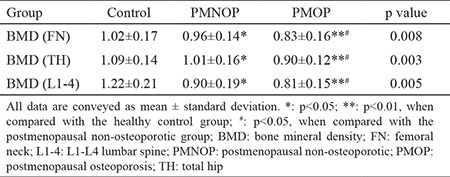
Comparison of bone mineral density at the femoral neck, total hip, and lumbar spine among postmenopausal non-osteoporotic, postmenopausal osteoporosis, and healthy control groups (bone mineral density unit: g/cm^2^)

**Table 3:**
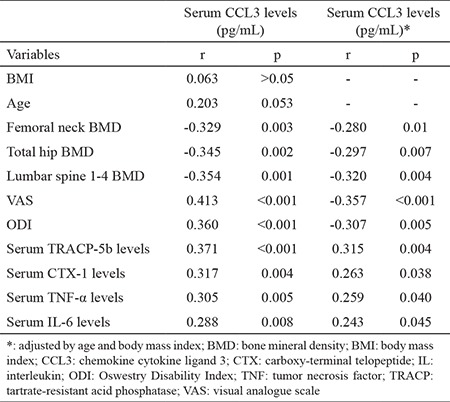
Correlation of serum chemokine cytokine ligand 3 concentrations with other indices in postmenopausal osteoporosis women adjusted by age and body mass index

**Table 4:**
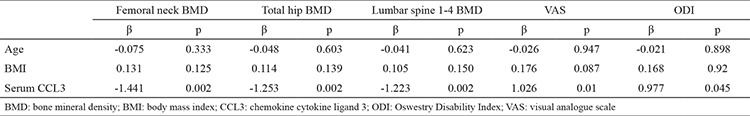
Multivariate linear regression for age, BMI and serum CCL3 with BMD ,VAS and ODI

## References

[1] Black DM, Rosen CJ (2016). Clinical Practice. Postmenopausal Osteoporosis. N Engl J Med.

[2] Pisani P, Renna MD, Conversano F, Casciaro E, Di Paola M, Quarta E, et al (2016). Major osteoporotic fragility fractures: risk factor updates and societal impact. World J Orthop.

[3] Compston J (2016). Osteoporosis: advances in risk assessment and management. Clin Med (Lond).

[4] Pietschmann P, Rauner M, Sipos W, Kerschan-Schindl K (2009). Osteoporosis: an age-related and gender-specific disease a mini-review. Gerontology.

[5] Abrahamsen B, van Staa T, Ariely R, Olson M, Cooper C (2009). Excess mortality following hip fracture: a systematic epidemiological review. Osteoporos Int.

[6] Chan CK, Mason A, Cooper C, Dennison E (2016). Novel advances in the treatment of osteoporosis. Br Med Bull.

[7] Schweser KM, Crist BD (2017). Osteoporosis: a discussion on the past 5 years. Curr Rev Musculoskelet Med.

[8] Fan YL, Peh WC (2016). Radiology of Osteoporosis: Old and New Findings. Semin Musculoskelet Radiol.

[9] Shetty S, Kapoor N, Bondu JD, Thomas N, Paul TV (2016). Bone turnover markers: Emerging tool in the management of osteoporosis. Indian J Endocrinol Metab.

[10] Eastell R, Hannon RA (2008). Biomarkers of bone health and osteoporosis risk. Proc Nutr Soc.

[11] Legler DF, Thelen M (2016). Chemokines: Chemistry, Biochemistry and Biological Function. Chimia (Aarau).

[12] Fu R, Liu H, Zhao S, Wang Y, Li L, Gao S, et al (2014). Osteoblast inhibition by chemokine cytokine ligand3 in myeloma-induced bone disease. Cancer Cell Int.

[13] Baba T, Mukaida N (2014). Role of macrophage inflammatory protein (MIP)-1α/CCL3 in leukemogenesis. Mol Cell Oncol.

[14] Jämsen E, Kouri VP, Ainola M, Goodman SB, Nordström DC, Eklund KK, et al (2017). Correlations between macrophage polarizing cytokines, inflammatory mediators, osteoclast activity, and toll-like receptors in tissues around aseptically loosened hip implants. J Biomed Mater Res A.

[15] Kawao N, Tamura Y, Horiuchi Y, Okumoto K, Yano M, Okada K, et al (2015). The Tissue Fibrinolytic System Contributes to the Induction of Macrophage Function and CCL3 during Bone Repair in Mice. PLoS One.

[16] Kukita T, Nomiyama H, Ohmoto Y, Kukita A, Shuto T, Hotokebuchi T, et al (1997). Macrophage inflammatory protein-1 alpha (LD78) expressed in human bone marrow: its role in regulation of hematopoiesis and osteoclast recruitment. Lab Invest.

[17] Fu R, Liu H, Zhao S, Wang Y, Li L, Gao S, et al (2014). Osteoblast inhibition by chemokine cytokine ligand3 in myeloma-induced bone disease. Cancer Cell Int.

[18] World Health Organization (1994). Assessment of fracture risk and its application to screening for postmenopausal osteoporosis: report of a WHO Study Group. World Health Organ Tech Rep Ser.

[19] Hawker GA, Mian S, Kendzierska T, French M (2011). Measures of adult pain: Visual Analog Scale for Pain (VAS Pain), Numeric Rating Scale for Pain (NRS Pain), McGill Pain Questionnaire (MPQ), Short-Form McGill Pain Questionnaire (SF-MPQ), Chronic Pain Grade Scale (CPGS), Short Form-36 Bodily Pain Scale (SF-36 BPS), and Measure of Intermittent and Constant Osteoarthritis Pain (ICOAP). Arthritis Care Res (Hoboken).

[20] Fairbank JC, Pynsent PB (2000). The Oswestry disability index. Spine (Phila Pa 1976).

[21] Charles JF, Nakamura MC (2014). Bone and the innate immune system. Curr Osteoporos Rep.

[22] Zupan J, Jeras M, Marc J (2013). Osteoimmunology and the influence of proinflammatory cytokines on osteoclasts. Biochem Med (Zagreb).

[23] Yoshie O, Imai T, Nomiyama H (2001). Chemokines in immunity. Adv Immunol.

[24] Kim MS, Magno CL, Day CJ, Morrison NA (2006). Induction of chemokines and chemokine receptors CCR2b and CCR4 in authentic human osteoclasts differentiated with RANKL and osteoclast like cells differentiated by MCP-1 and RANTES. J Cell Biochem.

[25] Kukita T, Kukita A, Harada H, lijima T (1997). Regulation of osteoclastogenesis by antisense oligodeoxynucleotides specific to zinc finger nuclear transcription factors Egr-1 and WT1 in rat bone marrow culture system. Endocrinology.

[26] Ohtori S, Akazawa T, Murata Y, Kinoshita T, Yamashita M, Nakagawa K, et al (2010). Risedronate decreases bone resorption and improves low back pain in postmenopausal osteoporosis patients without vertebral fractures. J Clin Neurosci.

[27] Cockerill W, Lunt M, Silman AJ, Cooper C, Lips P, Bhalla AK, et al (2004). Health-related quality of life and radiographic vertebral fracture. Osteoporos Int.

[28] Fechtenbaum J, Cropet C, Kolta S, Horlait S, Orcel P, Roux C (2005). The severity of vertebral fractures and health-related quality of life in osteoporotic postmenopausal women. Osteoporos Int.

[29] Iwamoto J, Takeda T, Sato Y, Uzawa M (2004). Effects of alendronate on metacarpal and lumbar bone mineral density, bone resorption, and chronic back pain in postmenopausal women with osteoporosis. Clin Rheumatol.

[30] Llorián-Salvador M, González-Rodríguez S, Lastra A, Fernández-García MT, Hidalgo A, Menéndez L, et al (2016). Involvement of CC Chemokine Receptor 1 and CCL3 in Acute and Chronic Inflammatory Pain in Mice. Basic Clin Pharmacol Toxicol.

[31] Teodorczyk-Injeyan JA, Triano JJ, McGregor M, McGregor M, Woodhouse L, Injeyan HS (2011). Elevated production of inflammatory mediators including nociceptive chemokines in patients with neck pain: a cross sectional evaluation. J Manipulative Physiol Ther.

[32] Hayman AR, Macary P, Lehner PJ, Cox TM (2001). Tartrate-resistant acid phosphatase (Acp 5): identification in diverse human tissues and dendritic cells. J Histochem Cytochem.

[33] Hayman AR, Dryden AJ, Chambers TJ, Warburton MJ (1991). Tartrate-resistant acid phosphatase from human osteoclastomas is translated as a single polypeptide. Biochem J.

[34] Wu Y, Lee JW, Uy L, Abosaleem B, Gunn H, Ma M (2009). Tartrate-resistant acid phosphatase (TRACP 5b): a biomarker of bone resorption rate in support of drug development: modification, validation and application of the BoneTRAP kit assay. J Pharm Biomed Anal.

[35] Vasikaran S, Eastell R, Bruyere O, Foldes AJ, Garnero P, Griesmacher A, et al (2011). Markers of bone turnover for the prediction of fracture risk and monitoring of osteoporosis treatment: a need for international reference standards. Osteoporos Int.

[36] Johansson H, Odén A, Kanis JA, McCloskey EV, Morris HA, Cooper C, et al (2014). A meta-analysis of reference markers of bone turnover for prediction of fracture. Calcif Tissue Int.

[37] Obrant KJ, Ivaska KK, Gerdhem P, Alatalo SL, Pettersson K, Väänänen HK (2005). Biochemical markers of bone turnover are influenced by recently sustained fracture. Bone.

[38] Kawana K, Takahashi M, Hoshino H, Kushida K (2002). Comparison of serum and urinary C-terminal telopeptide of type I collagen in aging, menopause and osteoporosis. Clin Chim Acta.

[39] Tella SH, Gallagher JC (2014). Prevention and treatment of postmenopausal osteoporosis. J Steroid Biochem Mol Biol.

[40] Brincat SD, Borg M, Camilleri G, Calleja-Agius J (2014). The role of cytokines in postmenopausal osteoporosis. Minerva Ginecol.

